# Evolutionary transition between invertebrates and vertebrates via methylation reprogramming in embryogenesis

**DOI:** 10.1093/nsr/nwz064

**Published:** 2019-05-24

**Authors:** Xiaocui Xu, Guoqiang Li, Congru Li, Jing Zhang, Qiang Wang, David K Simmons, Xuepeng Chen, Naveen Wijesena, Wei Zhu, Zhanyang Wang, Zhenhua Wang, Bao Ju, Weimin Ci, Xuemei Lu, Daqi Yu, Qian-fei Wang, Neelakanteswar Aluru, Paola Oliveri, Yong E Zhang, Mark Q Martindale, Jiang Liu

**Affiliations:** 1 CAS Key Laboratory of Genome Sciences and Information, Beijing Institute of Genomics, Chinese Academy of Sciences, Beijing 100101, China; 2 University of Chinese Academy of Sciences, Beijing 100029, China; 3 Institute of Apiculture Research, Chinese Academy of Agriculture Sciences, Beijing 100093, China; 4 Whitney Laboratory for Marine Bioscience, University of Florida, FL 32080, USA; 5 College of Life Sciences, Yantai University, Yantai 265600, China; 6 CAS Key Laboratory of Genomics and Precision Medicine, Beijing Institute of Genomics, Chinese Academy of Sciences, Beijing 100101, China; 7 Institute of Zoology, Chinese Academy of Sciences, Beijing 100101, China; 8 Biology Department, Woods Hole Oceanographic Institution, MA 02543, USA; 9 Departments of Genetics, Evolution and Environment, and Cell and Developmental Biology, University College London, London WC1E 6BT, UK; 10 CAS Center for Excellence in Animal Evolution and Genetics, Chinese Academy of Sciences, Kunming 650223, China

**Keywords:** DNA methylation, evolution, development, reprogramming

## Abstract

Major evolutionary transitions are enigmas, and the most notable enigma is between invertebrates and vertebrates, with numerous spectacular innovations. To search for the molecular connections involved, we asked whether global epigenetic changes may offer a clue by surveying the inheritance and reprogramming of parental DNA methylation across metazoans. We focused on gametes and early embryos, where the methylomes are known to evolve divergently between fish and mammals. Here, we find that methylome reprogramming during embryogenesis occurs neither in pre-bilaterians such as cnidarians nor in protostomes such as insects, but clearly presents in deuterostomes such as echinoderms and invertebrate chordates, and then becomes more evident in vertebrates. Functional association analysis suggests that DNA methylation reprogramming is associated with development, reproduction and adaptive immunity for vertebrates, but not for invertebrates. Interestingly, the single HOX cluster of invertebrates maintains unmethylated status in all stages examined. In contrast, the multiple HOX clusters show dramatic dynamics of DNA methylation during vertebrate embryogenesis. Notably, the methylation dynamics of HOX clusters are associated with their spatiotemporal expression in mammals. Our study reveals that DNA methylation reprogramming has evolved dramatically during animal evolution, especially after the evolutionary transitions from invertebrates to vertebrates, and then to mammals.

## INTRODUCTION

The invertebrate-to-vertebrate transition was a major event during the evolution of the animal kingdom. Vertebrates and invertebrates have several major morphological transitions. One main difference between vertebrates and invertebrates is that vertebrates have a backbone or spinal column, and invertebrates do not. In addition, vertebrates have an endoskeleton that is comprised of mineralized tissue in the form of bone and cartilage, while the majority of invertebrates have a non-cartilaginous exoskeleton. In addition, the central nervous system develops at the dorsal side of the vertebrate body, but ventrally in insects and many other invertebrates [1]. However, the underlying molecular mechanisms corresponding to these huge morphological differences during the invertebrate-to-vertebrate transition are virtually unknown.

At the molecular level, it has been found that whole-genome duplication (WGD) occurs during invertebrate-to-vertebrate transition [2]. Ohno stressed the contribution of gene duplication to the invertebrate-to-vertebrate transition [2]. The existence of multiple copies for many types of genes in vertebrate genomes support the hypothesis of WGD, such as the well-known HOX gene clusters. WGDs are believed to have provided the innovation and raw material for the origination of vertebrates [3]. However, WGDs also bring challenges for the genome to be properly regulated. Until now, the underlying regulatory mechanism has not been clearly identified.

Cytosine methylation (5mC) is an epigenetic modification that is largely restricted to CpG dinucleotides in animals [4]. CpG methylation serves multiple critical functions in the regulation of gene expression, genomic imprinting, transposon silencing and X-chromosome inactivation [4–6]. The genomic distribution of CpG methylation diversifies largely between different clades [7]. In vertebrates such as zebrafish, mice and humans, CpG methylation occurs nearly throughout the entire genome with exceptions in CpG-rich regions such as CpG islands [8–10], whereas invertebrates either lack cytosine methylation modification, such as worms and fruit flies, or show ‘mosaic’ methylation patterns, such as sea anemone, honey bee and sea squirt [9,11].

Sperm and oocytes are highly distinct and specialized cell types. Although they equally contribute genomic DNA to the zygote, their epigenetic states are highly asymmetric [12]. In both zebrafish and mammals, the global extent and distribution of 5mC in sperm is very different from that in oocytes [13–18]. The asymmetric methylomes of sperm and oocytes are then reprogrammed to equivalent states soon after fertilization. However, zebrafish and mammals have remarkably different reprogramming strategies [12]. In zebrafish, the paternal methylome is stably inherited, whereas the maternal methylome undergoes substantial remodeling to match the paternal methylome [14,15]. In contrast, in mammals, both parental genomes undergo extensive genome-wide demethylation [16–20]. These studies show that even between vertebrates, DNA methylation reprogramming strategies are surprisingly different. The different strategies may reflect the underlying developmental programs of mammals and fish. Therefore, we are curious about the methylation dynamics in invertebrates, which have dramatically different body plans.

HOX genes have a wide phylogenetic distribution within metazoans [21,22] and control morphologies on the main body axis of nearly all metazoans [23,24]. It is believed that changes in the HOX code might be causative for evolutionary novelties [25]. Invertebrates only have one set of HOX genes [22]; in contrast, vertebrates have multiple sets of HOX genes, which enable the increased morphological complexity of vertebrates [22,26,27]. Previous studies have illustrated that all HOX gene clusters in zebrafish are initially unmethylated in sperm and hypermethylated in oocytes, and eventually reprogram to an unmethylated pattern upon the mid-blastula transition (MBT) stage, when cell differentiation and segmentation are initiated [14,15]. It remains unclear whether and how DNA methylation plays roles in regulating HOX clusters before and after WGD.

To address these questions, we compared DNA methylomes for sperm, oocytes and early embryos from four invertebrate and three vertebrate species, and systematically analyzed the reprogramming of parental methylomes during metazoan evolution. Our findings have major implications for our understanding of the evolutionary transitions from invertebrates to vertebrates, and then to mammals, from the viewpoint of DNA methylation reprogramming during embryogenesis.

## RESULTS

### Single-base resolution DNA methylomes of gametes and early embryos in invertebrates

**Figure 1.**
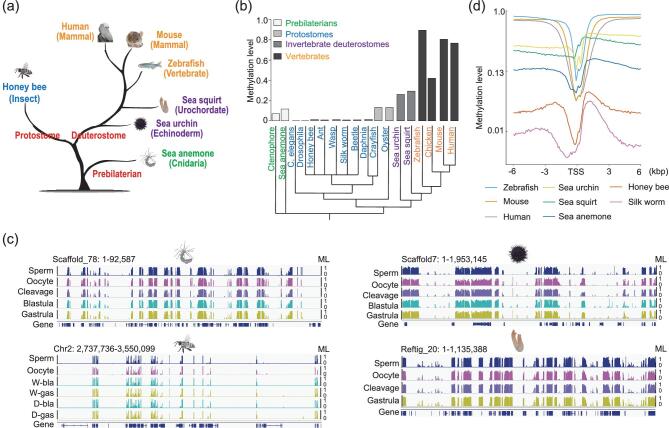
Conservation and divergence of global methylation patterns in animals. (a) Evolutionary tree of animal species used in this study. (b) Global average methylation levels across different animals. Cluster under bars represents evolutionary tree of animals which is derived from the Time Tree (http://timetree.org/). (c) Genomic snapshots (IGV) displaying mosaic methylation pattern in invertebrates. ‘W’ means worker bee. ‘D’ means drone bee. ‘bla’ means blastoderm. ‘gas’ represents ‘gastrula’. Vertical line height indicates the methylation level (ML). (d) Variation of methylation levels across 6 kb upstream and downstream of transcription start sites (TSSs) in sperm (methylation levels were calculated for every 100-bp bin).

To investigate the evolution of inheritance and reprogramming of parental methylation patterns, we performed whole-genome bisulfite sequencing (WGBS) to get DNA methylomes of sperm, oocytes and early embryos from sea anemone (*Nematostella vectensis*), honey bee (*Apis mellifera*), sea urchin (*Strongylocentrotus purpuratus*) and sea squirt (*Ciona savignyi*) (Supplementary Table S1). Previous studies have reported methylomes for the somatic tissues of sea anemone, honey bee and sea squirt at single-base resolution. The global methylation levels of our data are similar to those studies (Supplementary Table S1) [9,11]. We also included the published methylomes of gametes and early embryos from three vertebrates, including zebrafish (*Danio rerio*), mouse (*Mus musculus*) and human (*Homo sapiens*) [14,18,28]. We used these species, representing major animal branches, to explore the conservation and divergence of DNA methylation during evolution (Fig. 1a).

First, we explored the global methylation levels of sperm or somatic tissues in animals, including our data and the previously published data (Fig. 1b). The genomes of pre-bilaterians, such as sea anemone and ctenophore [29], have low global methylation levels around 0.10 (the methylation level is 0.11 and 0.08 for sea anemone and ctenophore, respectively, green branches). Protostomes are either missing DNA methylation due to a lack of DNA methyltransferases, such as *Drosophila* and *Caenorhabditis elegans* [9,11], or have rare DNA methylation abundance (methylation level ∼0.01) as seen in honey bee, ant [30], wasp [31], silkworm [32], beetle [33] and daphnia, or have methylation levels ∼0.15 such as marbled crayfish [34] and oyster [35] (Fig. 1b, blue branches). The methylation levels are ∼0.25–0.30 for invertebrate deuterostome sea urchin and sea squirt (Fig. 1b, purple branches). For vertebrates, global CpGs show medium levels of methylation (0.42) in chicken [36], or are highly methylated (methylation level > 0.75) in zebrafish and mammals (Fig. 1b, yellow branches). These observations clearly show the divergences of DNA methylation across animals.

Second, we observed a mosaic characteristic of invertebrate methylomes for both gametes and embryos, which means that the patterns of their genomes are composed of highly methylated regions interspersed with unmethylated regions (Fig. 1c). This observation is consistent with previous studies [9,11]. Our data further show that the mosaic pattern persists throughout the gametes and early embryos in sea anemone, honey bee, sea urchin and sea squirt (Fig. 1c).

Third, we plotted the fraction of all CpGs with different methylation levels in invertebrates, showing that CpGs have a bimodal distribution where they are either fully methylated or unmethylated in all stages (Supplementary Fig. S1a), which is consistent with the previous observations in vertebrates [8,14]. An inverse relationship between methylation levels and CpG densities in all invertebrates was also observed (Supplementary Fig. S1b), which is also consistent between invertebrates and vertebrates.

Fourth, we showed that promoters are consistently hypomethylated across all of the species studied, in line with previous reports [8,14,15,18] (Fig. 1d). Previous studies have shown that methylation in somatic cells preferentially targets gene body regions but not transposable elements in invertebrates, whereas vertebrates have methylation throughout the genome except for CpG islands [9,11]. Our data confirm this observation in both gametes and embryos in all invertebrates (Supplementary Fig. S2).

Taken together, our data expend previous knowledge about invertebrate methylomes in different cell types and species, and show the divergence and conservation across animals.

### Reprogramming of DNA methylomes during early embryogenesis in different species

**Figure 2.**
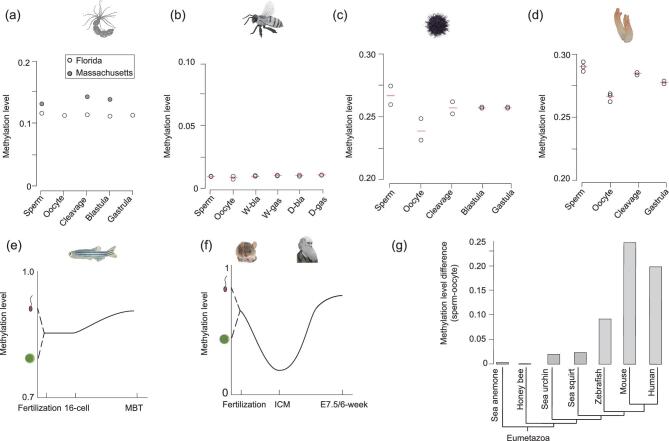
Evolution of methylation dynamic during early embryogenesis in animals. (a)–(d) The dynamics of the average methylation levels in sea anemone (a), honey bee (b), sea urchin (c) and sea squirt (d). (e) Graphic model of DNA methylation dynamics during zebrafish early embryogenesis from previous data. ‘MBT’ means midblastula transition. (f) Graphic model of the DNA methylation dynamics during mammalian early embryogenesis from previous data. ‘E7.5’ means mouse embryos 7.5 days after fertilization. ‘6-week’ means human embryos 6-weeks after fertilization. (g) Methylation level differences between sperm and oocytes for seven species. Tree topology is from the Time Tree (http://timetree.org/).

The reprogramming of parental methylomes during early embryogenesis has been well characterized in zebrafish and mammals; however, little is known about the reprogramming of DNA methylation in invertebrates. Here, we investigated the dynamic changes of DNA methylomes of gametes and early embryos for sea anemone, honey bee, sea urchin and sea squirt. For each stage, at least two independent biological replicates were sequenced for honey bee, sea urchin and sea squirt. The methylation levels were highly correlated between replicates (Fig. 2a–d and Supplementary Table S2). The average bisulfite conversion rate (Supplementary Table S1) was 99.26% and the standard deviation was 0.14%, which indicates that the quality of our libraries is good.

For sea anemone, two methylome data sets of gametes and early embryos sampled from Massachusetts and Florida were used in this study (Fig. 2a). The methylation levels were slightly different between these two data sets, which may have been caused by variance in their genetic backgrounds and/or their living environments. Interestingly, the global methylation levels of sea anemone sperm and oocytes were similar, and differed from those in mammals and zebrafish. Moreover, the methylation levels of early embryos at different stages were also similar to those of the gametes in sea anemone (Fig. 2a). In addition, no significant differences in methylation level were found for various genomic elements during sea anemone embryogenesis (Supplementary Fig. S3). These results indicate that there is no significant dynamic of DNA methylation during early development in sea anemone, which is different from that in zebrafish and mammals.

Recently, a study used sequencing of Methyl-CpG-binding domain (MBD)-biotin-based selection of CpG-methylated DNA, which can only cover a limited proportion of CpGs, to analyze the sperm, oocytes and adult drones of honey bee [37]. In our study, we provided genome-wide maps and compared the methylation levels of sperm, oocytes and early embryos of workers and drones in honey bee. Our data show that the methylation levels of all stages examined are ∼0.01, which are similar to the levels of previously published methylomes in honey bee [11] (Fig. 2b and Supplementary Fig. S3).

In contrast to the situation seen in sea anemone and honey bee, dynamics of DNA methylation levels were observed from gametes to early embryos in both sea urchin and sea squirt (Fig. 2c and d). Similar results were also observed for the methylation levels of various genomic elements (Supplementary Fig. S3), indicating that methylome reprogramming is present in both sea urchin and sea squirt. Previous studies have shown the global methylation differences between zebrafish gametes and the inheritance of the sperm methylome by early zebrafish embryos [14,15] (Fig. 2e). In mammals, the dynamics of methylation are even more dramatic, with genome-wide demethylation occurs during early embryogenesis [16–18,20,38] (Fig. 2f). Our data also showed that the methylation levels of sperm were higher than those of oocytes in both sea urchin (Fig. 2c) and sea squirt (Fig. 2d). Following this direction, we compared the methylation levels of sperm and oocytes for all seven species, and revealed that differences in methylation levels between sperm and oocytes increase through deuterostome and chordate evolution (Fig. 2g).

Taken together, parental methylomes are almost identical and remain stable during embryogenesis in pre-bilaterians such as cnidarians and in protostomes such as insects. Reprogramming of parental methylomes is clearly present in echinoderms and invertebrate chordates, and became more evident during vertebrate evolution.

### Absence of non-CpG methylation in the oocytes of invertebrates

Previous studies have unveiled the presence of non-CpG methylation in the oocytes of both mouse and human [13,16,18,39]. In contrast, non-CpG methylation has not been detected in zebrafish oocytes [14,15]. It remains unknown whether non-CpG methylation in oocytes is unique to mouse and human. Here, we examined non-CpG methylation in invertebrates. Global non-CpG methylation levels were calculated by subtracting the bisulfite non-conversion rates from the average methylation levels (see Supplementary Data). Our data show that the methylation levels of non-CpGs are not significant in the oocytes and early embryos from sea anemone, honey bee, sea urchin and sea squirt (Supplementary Fig. S4), and are similar to that of zebrafish. Therefore, our data suggest that non-CpG methylation in oocytes is unique to mammals.

### Evolution of promoter methylation reprogramming during embryogenesis

**Figure 3.**
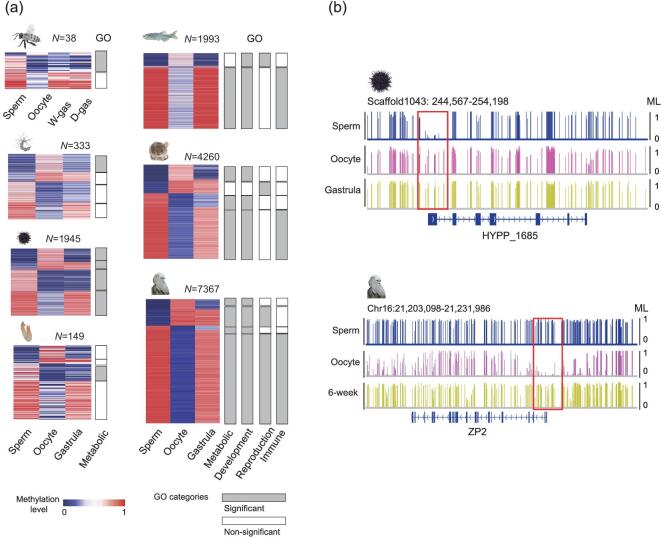
Evolution of promoter reprogramming in animals. (a) Heatmaps of differentially methylated promoters between sperm and oocytes across different species. Gene ontology (GO) enrichment of genes with differentially methylated promoters was performed. The color key from blue to pink indicates the DNA methylation levels (MLs) from low to high, respectively. (b) Genomic snapshot shows reprogramming of promoters in sea urchin and human. Red boxes highlight the promoter regions.

It has been reported that DNA methylation in promoters can regulate gene expression [40,41], while the roles of DNA methylation in genic regions are uncertain. Therefore, we focused on promoter methylation to examine the potential impact of methylation reprogramming on animal evolution. We performed gene ontology analyses of differentially methylated promoters (DMPs) between sperm and oocytes (see Supplementary Data). DMPs identified in honey bee, sea anemone and sea squirt were very limited. Using the short list of genes, only metabolism category-related pathways could be found (Fig. 3a, Supplementary Table S3). Nevertheless, our data suggest that the potential regulation of DNA methylation reprogramming in these species is very limited. Our data also show that genes with DMPs are enriched in general metabolic pathways in sea urchin (Fig. 3a and b, and Supplementary Table S3), which is similar to the situations in other invertebrates. In zebrafish and mammals, genes with DMPs are enriched in not only metabolic pathways, but also many developmental and reproductive pathways (Fig. 3a and b, and Supplementary Table S3), suggesting that DNA methylation reprogramming is associated with vertebrate embryonic development and reproduction. Moreover, the enriched category of the adaptive immune system, a unique system in jawed vertebrates [3], can also be observed in vertebrates (Fig. 3a). Together, our analysis suggests that DNA methylation reprogramming of promoters is associated with development, reproduction and adaptive immunity in zebrafish and mammals, but not invertebrates.

CpG density is generally anti-correlated with DNA methylation level [8]; however, many oocyte promoters do not follow the rule of anti-correlation between CpG density and methylation level [15]. This correlation remains elusive in oocytes of other animals. Therefore, we plotted scatterplots for CpG densities and methylation levels for all promoters, and calculated the Pearson correlation coefficient (PCC) for each sample in different species (Supplementary Fig. S5). Our data showed that the anti-correlation patterns between methylation levels and CpG densities were similar between oocytes and sperm/gastrula in invertebrates. However, in mammals, a large proportion of low-CpG promoters in oocytes showed low or medium methylation levels, which differed to the sperm/gastrula (Supplementary Fig. S5, red boxes). The PCC of the oocytes was also the lowest among different cell types and tissues in human (Supplementary Fig. S6a). Further analysis showed that, in vertebrates, the majority of oocyte-specific hypomethylated promoters (versus sperm) had low CpG densities (Supplementary Fig. S6b), while most oocyte-specific hypermethylated promoters had high CpG densities (Supplementary Fig. S6c). These results indicate that a significant proportion of promoters in the oocytes of mammals do not follow the rule of anti-correlation between CpG density and methylation level.

### The reprogramming of HOX genes during animal evolution

**Figure 4.**
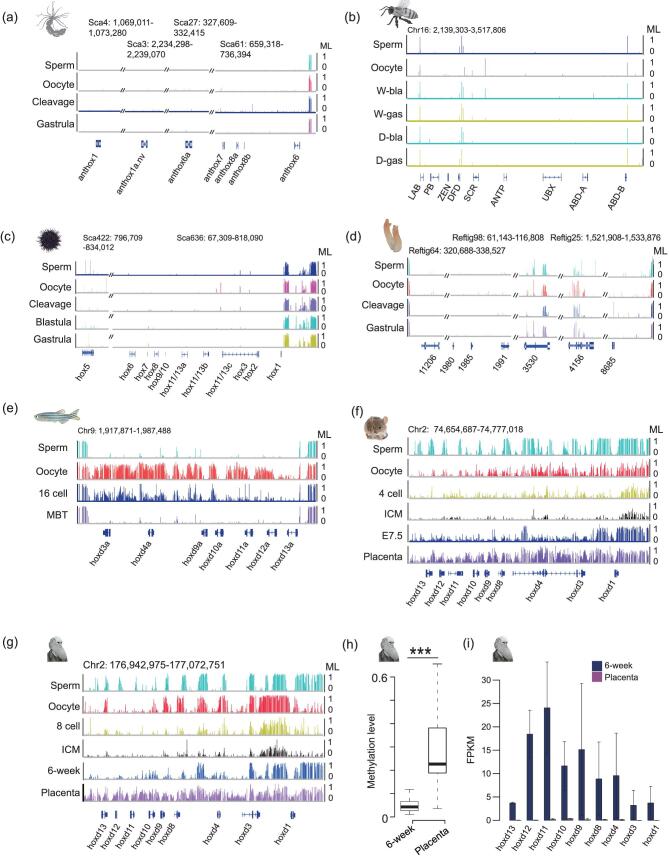
Methylation of the HOX gene clusters in different taxa. (a)–(d) Genomic snapshots representing methylation of the HOX gene clusters in sperm, oocytes and early embryos of sea anemone (a), honey bee workers and drones (b), sea urchin (c) and sea squirt (d). Oblique lines represent regions of the HOX cluster that are non-contiguous or interrupted. (e)–(g) Genomic snapshots show methylation dynamics of HOX gene clusters in zebrafish (e), mouse (f) and human (g). (h) Boxplots show the promoter methylation levels (MLs) of HOX genes between 6-week embryos and placenta in human. *P* value was calculated by paired Wilcoxon signed-rank test. ***P < 0.001. (i) Normalized expression levels of HOXD genes in human 6-week embryos and placenta. FPKM stands for fragments per kilobase of transcript per million mapped reads.

HOX genes are a subset of homeobox genes that play crucial roles in segmentation during development and the evolution of the metazoan body plan [23,24]. Our previous study showed that all HOX gene clusters in zebrafish are initially unmethylated in sperm and hypermethylated in oocytes, and eventually reprogram to a unmethylated pattern during the MBT stage [14] (Fig. 4e) when cell differentiation and segmentation are initiated [42]. However, the methylation reprogramming of HOX clusters in animals beyond zebrafish has not been addressed. Here, we checked the methylation dynamics of HOX clusters in different species. HOX genes are unmethylated in the somatic tissues of daphnia, silkworm and oyster (Supplementary Fig. S7a–c). At genic regions, the status of HOX genes is different from those of housekeeping genes, which are usually hypermethylated (Supplementary Fig. S8). Importantly, our data showed that HOX genes, including both genic regions and promoters, are generally unmethylated and show no dynamics in gametes and early embryos in invertebrates, including sea anemone (Fig. 4a), honey bee (Fig. 4b), sea urchin (Fig. 4c) and sea squirt (Fig. 4d), which differs from that seen in zebrafish. The differences are more distinguished when compared to mammals, both in terms of methylation pattern and reprogramming. Our data showed that HOX gene clusters in mammalian sperm and oocytes are not uniformly methylated or unmethylated (Fig. 4f and 4g, and Supplementary Fig. S9a–c, e–g). Moreover, all HOX clusters are reprogrammed to be unmethylated in Inner Cell Mass (ICM) stage when genome-wide demethylation is completed. The unmethylated status of HOX clusters is mostly maintained in mouse E7.5 embryos or human 6-week embryos when segmentation takes place during mammalian development (Fig. 4f and g, and Supplementary Fig. S9a–c, e–g). Very interestingly, HOX clusters are partially methylated in the placenta of both mouse and human, where segmentation is not desired (Fig. 4f and g, and Supplementary Fig. S9a–c, e–g). This is further demonstrated by the fact that most HOX gene promoters are significantly hypermethylated in placenta compared to in 6-week/E7.5 embryos (Fig. 4h and Supplementary Fig. S9d). Further gene expression analysis demonstrated that most HOX genes are expressed in human 6-week embryos, but not in placenta (Fig. 4i and Supplementary Fig. S9h–j), showing that DNA methylation anti-correlates with the expression of HOX genes in human 6-week embryos and placenta. We then checked the methylation patterns of HOX gene clusters in human organs using data from the roadmap project [43]. Methylation exhibits very different patterns among different organs (Supplementary Fig. S7d), which are distinguishable from the stable unmethylated state in tissues of invertebrates. In summary, in invertebrates with a single cluster of HOX genes, HOX genes are usually unmethylated and unprogrammed in gametes, early embryos and somatic tissues, suggesting that DNA methylation has no role in regulating invertebrate HOX genes. In contrast, duplicated HOX clusters of vertebrates undergo dramatic methylation reprogramming during embryogenesis that is associated with the spatiotemporal gene expression of HOX genes. We hypothesize that methylation reprogramming regulates vertebrate HOX clusters, which helps vertebrate genomes to achieve the goal of spatiotemporal expression of multiple HOX clusters.

In addition to HOX genes, other homeobox genes play important roles in embryonic patterning and cell differentiation [44]. Our analysis showed that a small proportion of non-HOX homeobox genes are hypermethylated or methylated to a medium level in gametes and early embryos in invertebrates, and that no dynamics are observed (Supplementary Fig. S10a). In contrast, we observed weak dynamics of methylation from oocytes to blastula/gastrula embryos in zebrafish. Notably, promoter regions of non-HOX homeobox genes were significantly hypermethylated in mammalian placenta compared with E7.5 or 6-week embryos (Supplementary Fig. S10a, b, c). The expression of non-HOX homeobox genes in placenta is also very limited compared to those in 6-week human embryos (Supplementary Fig. S10d), suggesting that promoter methylation anti-correlates with the expression of non-HOX homeobox genes in mammals.

### The reprogramming of key developmental signaling genes during animal evolution

Many signaling factors, such as FGF, Hedgehog, NOTCH, TGF-β and WNT, are usually conserved and play crucial roles in embryonic development [45]. Our data showed that both promoter and genic regions of almost all these genes were maintained in an unmethylated state, and showed no dynamics in invertebrates (Supplementary Fig. S11). In zebrafish, most promoters of such genes were unmethylated throughout embryogenesis, while genic regions maintained their hypermethylated states (Supplementary Fig. S11). In mammals, the promoter regions of most genes had unmethylated status in sperm, oocytes and embryos, while many genes showed significant reprogramming in genic regions (Supplementary Fig. S11). In general, such genes have hypomethylated genic regions and are rarely expressed in mammalian oocytes (Supplementary Fig. S12), which is in line with previous studies that have shown that gene body methylation positively correlates with gene expression in mammalian oocytes [13,16]. Such genes usually have unmethylated promoters in gametes and embryos, and show high expression in human 6-week embryos (Supplementary Fig. S12). In summary, our study shows a significant evolution of DNA methylation reprogramming of key developmental signaling factors.

## DISCUSSION

Here, we have systematically investigated the conservation and evolution of DNA methylation reprogramming during early embryogenesis in animals. Our results reveal that different clades use distinct strategies of DNA methylation reprogramming during embryogenesis and that not all species undergo methylation reprogramming (Table 1). Invertebrate genomes are devoid of DNA methylation or have a mosaic methylation pattern, which limits the dynamics of DNA methylation. In contrast, vertebrate methylomes are globally methylated, which can favor a large range of methylation reprogramming (Fig. 2). Global DNA methylation remodeling is vertebrate-specific, which can fit the need for complex regulation of vertebrate developmental process. Indeed, the dramatic evolution of DNA methylation reprogramming during the transition from invertebrates to vertebrates has enabled more precise regulation in development, reproduction and the origins of new genes, such as the adaptive immune system genes in vertebrates (Fig. 3). Therefore, we hypothesize that the evolution of DNA methylation reprogramming has helped the transition from invertebrates to vertebrates.

**Table 1.** Evolution of DNA methylation reprogramming.

	Methylation level	Early embryo reprogramming	Gene ontology enrichment	Genome-wide demethylation
Cnidarian	∼0.10	No	Metabolism	No
Insect	∼0.01	No	Metabolism	No
Invertebrate deuterostome	∼0.25	Minor	Metabolism	No
Vertebrate	>0.4	Moderate	Metabolism, development, adaptive immune, reproduction	No
Mammal	>0.7	Dramatic	Metabolism, development, adaptive immune, reproduction	Yes

Genome-wide demethylation only occurs during mammalian embryogenesis (Fig. 2). One important function for DNA methylation is the control of imprinted genes, which is only found in placental mammals [46,47]. In the animal kingdom, most animals undergo fertilization and embryos develop externally [48]. In placental mammals, embryos develop with a placenta within the mother's uterus. It is believed that genomic imprinting is a battle between parents to control the size and nourishment of an embryo within the mother's uterus [47,49]. In contrast, all other animals, which develop without a placenta, do not need imprinting to control fetus size and nutrition transfer. Therefore, genome-wide demethylation in mammals may be necessary for the generation of imprinting, which may have been a major innovation in the evolutionary transition to placental viviparity.

It has been suggested that genome duplication was major driver in the origin of vertebrates [50]. Genome duplication has led to the duplication of HOX genes [22], as well as the emergence of many new genes [51]. HOX genes define the body plan via segmentation [21–24]. Invertebrates have only one cluster of HOX genes [22], and our data have shown that HOX genes are unmethylated throughout early embryogenesis in invertebrates (Fig. 4). It is probable that histone modification, non-coding RNA and other epigenetic modifications are enough to regulate the expression of one set of HOX genes in invertebrates. Vertebrates have multiple sets of HOX genes, which enable vertebrates to have more complicated body plans [22], but this larger number of HOX genes also brings challenges regarding the genome being able to express each individual HOX gene in the right place and at the right time. To deal with this issue, it seems that DNA methylation reprogramming in HOX gene clusters was added into vertebrate genomes to enable more elaborate spatiotemporal control of HOX gene expression. HOX genes are not expressed in oocytes or during the cleavage stages of embryos, and instead begin to be expressed when cell differentiation and segmentation starts [14]. To avoid ‘leaky’ expression of HOX genes in oocytes, HOX gene clusters are hypermethylated in zebrafish oocytes (Fig. 4e). Since gene expression in sperm is very limited, HOX genes are not expressed even though all HOX gene clusters are unmethylated in zebrafish sperm [14]. Zebrafish HOX genes reprogram to unmethylated states by the MBT stage (Fig. 4e), when cell differentiation and segmentation starts [14,15,42]. In mammals, HOX gene clusters are often partly methylated in sperm and oocytes (Fig. 4f and g, and Supplementary Fig. S9). Genome-wide demethylation erases all methylation in the blastocyst stage, when cell differentiation and segmentation starts in mammals. Segmentation does not occur in the placenta, which is consistent with our observation that all HOX gene clusters in the placenta are partially methylated (Fig. 4f and g, and Supplementary Fig. S9). In mammalian somatic tissues, only certain HOX genes maintain their unmethylated status in specific tissues (Supplementary Fig. S7d). Taken together, our data suggest that DNA methylation reprogramming in HOX genes plays an important role in vertebrates, enabling them to take advantage of duplicated HOX gene clusters by regulating their spatiotemporal expression.

Taken together, our data provide an epigenetic clue that increases our understanding of the invertebrate-to-vertebrate transition and also placental viviparity. Our study opens up a new view in the understanding of evolution and development.

## AVAILABILITY OF DATA AND MATERIALS

WGBS data for sea anemone, honey bee, sea urchin, sea squirt, daphnia and mouse placenta have been deposited in the Genome Sequence Archive (GSA) [52] at the BIG Data Center [53], Beijing Institute of Genomics (BIG), Chinese Academy of Sciences (CAS), under accession number CRA001225; they are publicly accessible at http://bigd.big.ac.cn/gsa. External data used in this study are shown in Table 2.

**Table 2.** External data used in this study.

Resource	Source	Database	Identifier
Human 6-week and placenta RNA, methylomes	Li *et al*. [28]	GSA	CRA000114
Human oocyte RNA	Yan *et al.* [54]	GEO	GSE36552
Zebrafish methylomes	Jiang *et al.* [14]	GEO	GSE44075
Mouse methylomes	Wang *et al.* [18]	GEO	GSE56697
Ctenophore methylome	Emily *et al.* [29]	SRA	SRR1981481
Oyster methylome	Wang *et al.* [35]	GEO	GSE40302
Silkworm methylome	Xiang *et al.* [32]	GEO	GSE18315
Ant methylome	Bonasio *et al.* [30]	GEO	GSE31577
Marbled crayfish methylome	Fanny *et al.* [34]	GEO	GSE112411
Wasp methylome	Wang *et al.* [31]	GEO	GSE43423
Chicken methylome	Mugal *et al.* [36]	GEO	GSE56639
Beetle methylome	Song *et al.* [33]	GEO	GSE 84253

## MATERIALS AND METHODS

The detailed methods and materials are available as Supplementary Data at *NSR* online.

## Supplementary Material

nwz064_Supplemental_Files
