# Supplementary material for: Evolutionary transition between invertebrates and vertebrates via methylation reprogramming in embryogenesis
Source: Natl Sci Rev. 2019 May 24;6(5):993–1003. doi: 10.1093/nsr/nwz064 (PMC8291442; doi:10.1093/nsr/nwz064)
Supplement: nwz064_Supplemental_Files [file nwz064_supplemental_files.zip › MethylationEvolution SupplementaryMaterials-2019-5-8.docx]

Supplementary Materials for

Comparative analysis of DNA methylation reprogramming during embryogenesis across metazoan

**This file includes:**

Materials and Methods

Figs. S1 to S12

Table S1

Legends for Table S2 and S3

References

**Other Supplementary Materials include the following:**

Table S2 and S3 (Excel files)

**Materials and Methods:**

**Sample collection**

Sea anemone: two set of sea anemone samples were collected from Massachusetts and Florida, respectively. The wild-type sea anemone lines were raised in standard laboratory conditions. We applied published methods and morphological stage criteria to collect gametes and embryos ^1,2,3^. Briefly, adult anemones were set up for spawning on the evening before the day of sampling. Sperm-containing water from male-only bowls was collected and possible somatic cell contamination was removed by a low-speed centrifugation. To collect embryos, the egg masses were fertilized with sperm-containing water. Egg masses and embryos were dejellyed at room temperature until the jelly was dissolved and washed several times to exclude contamination.

Honey bee: the wild-type honey bee lines were raised in standard laboratory conditions in Beijing, China. Sperm was collected using methods developed previously as part of artificial insemination technique ^4^. To collect semen, the abdomen of the drone was gently squeezed between two fingers resulting in the ejaculate to appear at the end of the endophallus. Sperm was washed by semen diluent and possible somatic cell contamination was removed by a low-speed centrifugation. We applied previously described methods and morphological stage criteria to sample unfertilized egg and embryos ^5,6^. To collect unfertilized egg or drone embryos, queen bees were allowed to lay eggs on drone combs in beehive for 30 minutes. To collect worker embryos, queen bees were allowed to lay eggs on worker combs in beehive for 30 minutes. Then the combs were taken out from beehive without queen bee. For unfertilized egg, samples are collected immediately. For embryos, the comb was incubated at 35℃to develop to blastoderm and gastrulation embryos, respectively. All samples were kept on ice and washed with chilled PBS to exclude contamination.

Sea urchin: the wild-type sea urchin lines were raised in standard laboratory conditions in California, US. Gametes and embryos were obtained by previously published methods^7^. Possible somatic cell contamination was removed by a low-speed centrifugation for sperm. Eggs and embryos were dejellyed and extensively washed.

Sea squirt: Wild animals were collected from the bay of Yantai, China. We followed established protocols ^8^ to obtain sea squirt gametes and embryos. Dechorionated eggs or embryos were extensively washed by swirling plate for more than ten times, which left the non-dechorionated eggs or embryos and cell debris behind. After washing, any eggs or embryos with somatic cells were removed by glass capillary under microscope.

Daphnia: The daphnia clones were maintained in laboratory at Massachusetts, US. Adult daphnia (20-25 individuals) were collected for isolation of genomic DNA.

Mouse placenta: placenta tissue was collected from full-term mouse.

**DNA Extraction**

Gametes, embryos and tissues were subjected to genomic DNA extraction by using QIAamp DNA Mini Kit (QIAGEN) following the protocol of DNA Purification from Tissues.

**WGBS Library Generation**

Genomic DNA spiked with 0.5% unmethylated Lambda DNA (Promega) was sonicated into 100-500bp. Fragmented DNA was then subjected to end repair, dA tailing, and adaptor ligation with the end repair enzyme mix (NEB), Klenow 3´-5´ exo- (NEB) and T4 DNA ligase (NEB), respectively. Adapter-ligated DNA was recovered by 2% agarose gel electrophoresis (Zymo Research). Bisulfite conversion was performed using the EZ DNA methylation-Gold kit (Zymo Research) according to the manufactory instruction. Bisulfite-treated DNA was then amplified with KAPA HiFi HotStart Uracil+ ReadyMix. Amplified DNA was purified by 2% agarose gel electrophoresis. DNA methylome libraries were sequenced on Illumina Hiseq2000 or Hiseq2500 sequencer. Each developmental stage has at least two biological replications, with exception for sea anemone oocyte.

**WGBS: Read Filtering, Alignment and Quantiﬁcation of methylation level for CpG sites**

WGBS data processing procedures were adapted from previously developed methods^9,10^ with minor modification.

Read ﬁltering: adapters and low-quality reads were trimmed with Trimmomatic (<http://www.usadellab.org/cms/index.php?page=trimmomatic>) with parameters “LEADING:3 TRAILING:3 SLIDINGWINDOW:4:15 MINLEN:36”. The paired-reads were discarded from following analysis if either end was shorter than 36 bases.

Alignment and post-alignment filtering: The reference genomes were downloaded from Ensembl (<http://www.ensembl.org/index.html>) and Ensembl Metazoa (<http://metazoa.ensembl.org/index.html>). The genome build for sea anemone, honey bee, sea urchin, sea squirt, zebrafish, mouse and human are ASM20922v1, GCA_000002195.1, Spur_3.1, CSAV2.0, zv9, mm10 and hg19, respectively. The lambda DNA genome was also included in the reference sequence as an extra chromosome for calculating bisulﬁte conversion rate. Filtered paired-end methylC-seq reads were mapped against the reference by Bismark ^11^. Duplicated reads were removed by deduplicate_bismark function of Bismark. Following deduplication, overlapped part of paired reads was trimmed from one end using clipOverlap function of bamUtil (<http://genome.sph.umich.edu/wiki/BamUtil:_clipOverlap>). The coverage of each cytosine was extracted from the clipOverlapped reads with bismark_methylation_extractor.

Quantiﬁcation of methylation level for CpG sites: for CpG i, deﬁne m_i_ as the number of reads showing methylation over position i (both strands). Deﬁne u_i_ as the number of reads showing lack of methylation over CpG i. The methylation level is estimated as m_i_/( m_i_ + u_i_), which is an estimate of the probability that CpG i is methylated in a molecule sampled randomly from the cell population. Because CpG methylation is symmetric, mi and ui include observations associated with the cytosines on both strands for the i-th CpG.

**Quantiﬁcation of average methylation level of CpGs in each sample:** average methylation level in each sample was measured as the sum of the methylation level of CpG cytosines, divided by the total number of CpG cytosines. To exclude the effect of bisulfite non-conversion, we adjusted average methylation level by subtracting the bisulfite non-conversion rate from the average methylation level. The bisulfite non-conversion rate is calculated as the methylation level in spiked-in unmethylated lambda DNA, which can indicate the failure of converting from un-methylated C to T as there is no methylated C in lambda DNA. The bisulfite conversion rate equals to 1 minus bisulfite non-conversion rate.

**Quantiﬁcation of average methylation level of CpGs in different genomic elements:** average methylation level of genomic elements was measured as the sum of the methylation level of CpG cytosines, divided by the total number of CpG cytosines that locate in those genomic elements. Promoters are defined as regions 2 kb upstream from TSSs (transcriptional start sites) for each gene. Only regions covered by at least 5 CpGs and each CpG site covered by at least five reads were considered for further analysis.

**Annotation datasets**

Gene annotations for invertebrates were downloaded from Ensembl (<http://www.ensembl.org/index.html>) and Ensembl Metazoa (<http://metazoa.ensembl.org/index.html>). Repeats of invertebrates were annotated using RepeatMasker^12^ with release 20130422 of the Genetic Information Research Institute nucleotide library ([www.girinst.org](http://www.girinst.org)). Gene annotations and repeat annotations for zebrafish, mouse and human were downloaded from UCSC table browser.

**Identiﬁcation of Differentially Methylated Promoters (DMPs)**

To identify DMPs between sperm and oocytes, we first performed Differentially Methylated Regions (DMRs) analysis using DSS package ^13^. The parameters for invertebrates and zebrafish were “delta=0.2, minCG=3, p.threshold=0.01, minlen=50”, and the parameters for mammals were “delta=0.3, minCG=5, minlen=100”. Promoters overlapped with DMRs were identified as DMPs. Gene Ontology (GO) analysis of genes with DMPs for each cluster was performed by using DAVID ^14^ for vertebrates and EASE ^15^ for invertebrates, respectively. GO terms with p value less than 0.1 were considered as statistically significant. GO terms hit by less than 5 genes were discarded in vertebrates, and terms hit by less than 2 genes were discarded in invertebrates.

**Quantiﬁcation of methylation level of non-CpGs:** The methylation level of each non-CpG(CHH and CHG, H means A, T, or C) context was determined as the sum of the methylation level of cytosines divided by the total number of cytosines in that context. We adjusted methylation level by subtracting the bisulfite non-conversion rate from the methylation level. The calculation of bisulfite non-conversion rate is described above.

**mRNA-Seq data analysis**

RNA-seq raw data of human were downloaded from GSE36552 and CRA000114，respectively. Low quality reads of RNA-seq and adaptor sequences were removed by Trimmomatic, followed by alignment with TopHat. The unique reads were used to calculate the fragments per kilobase of exon per million fragments mapped (FPKM) with Cufflinks v2.0.2 (http://cufflinks.cbcb.umd.edu).

**Sequencing data visualization**

DNA methylation levels are visualized as custom tracks in the Integrative Genomics Viewer genome browser (<http://software.broadinstitute.org/software/igv/>).

**Statistical analysis of sequencing datasets**

Statistical analyses and plots were implemented with R(<http://www.r-project.org>). Pearson Correlation Coefficient was calculated using the ‘cor’ function with default parameters. Wilcoxon signed-rank test (two-tailed) was performed using ‘wilcox.test’ function with parameters “paired=TRUE”. Fisher’s Exact Test was performed using the ‘fisher.test’ function with default parameters. BH-adjusted p values were calculated with ‘p.adjust’ function with “method=“BH””.


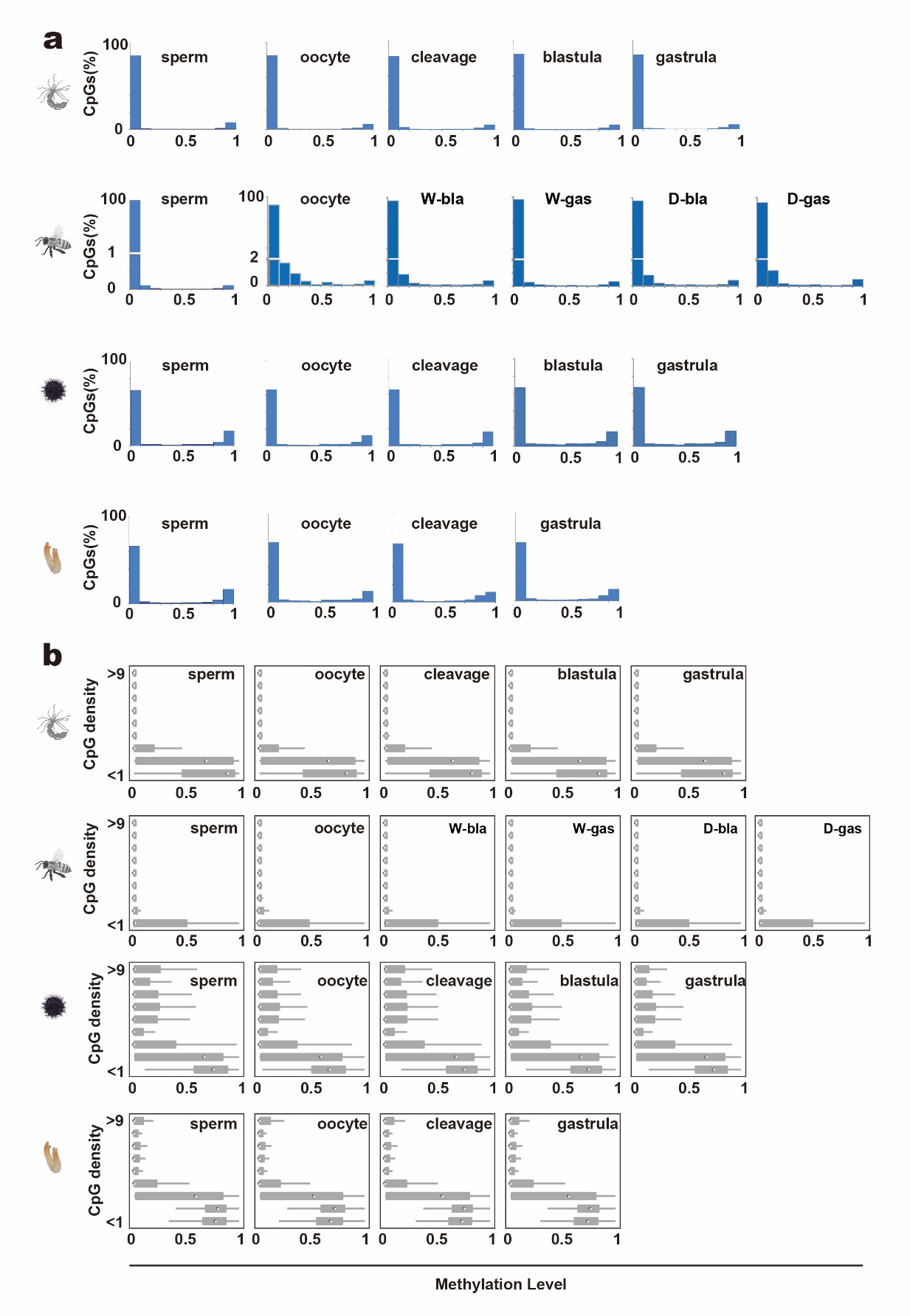


**Supplementary Fig. 1 |** **Bimodal distribution of methylation level and inverse correlation between CpG density and methylation level in invertebrates**

**(a)**, Fractions of all CpGs with different methylation level in gametes and early embryos of different animals. **(b)**, Box plots of methylation levels across CpG densities of 1000bp-tiles. Circle indicates the median, edges the 25^th^/75^th^ percentile and whiskers the 2.5^th^/97.5^th^ percentile.


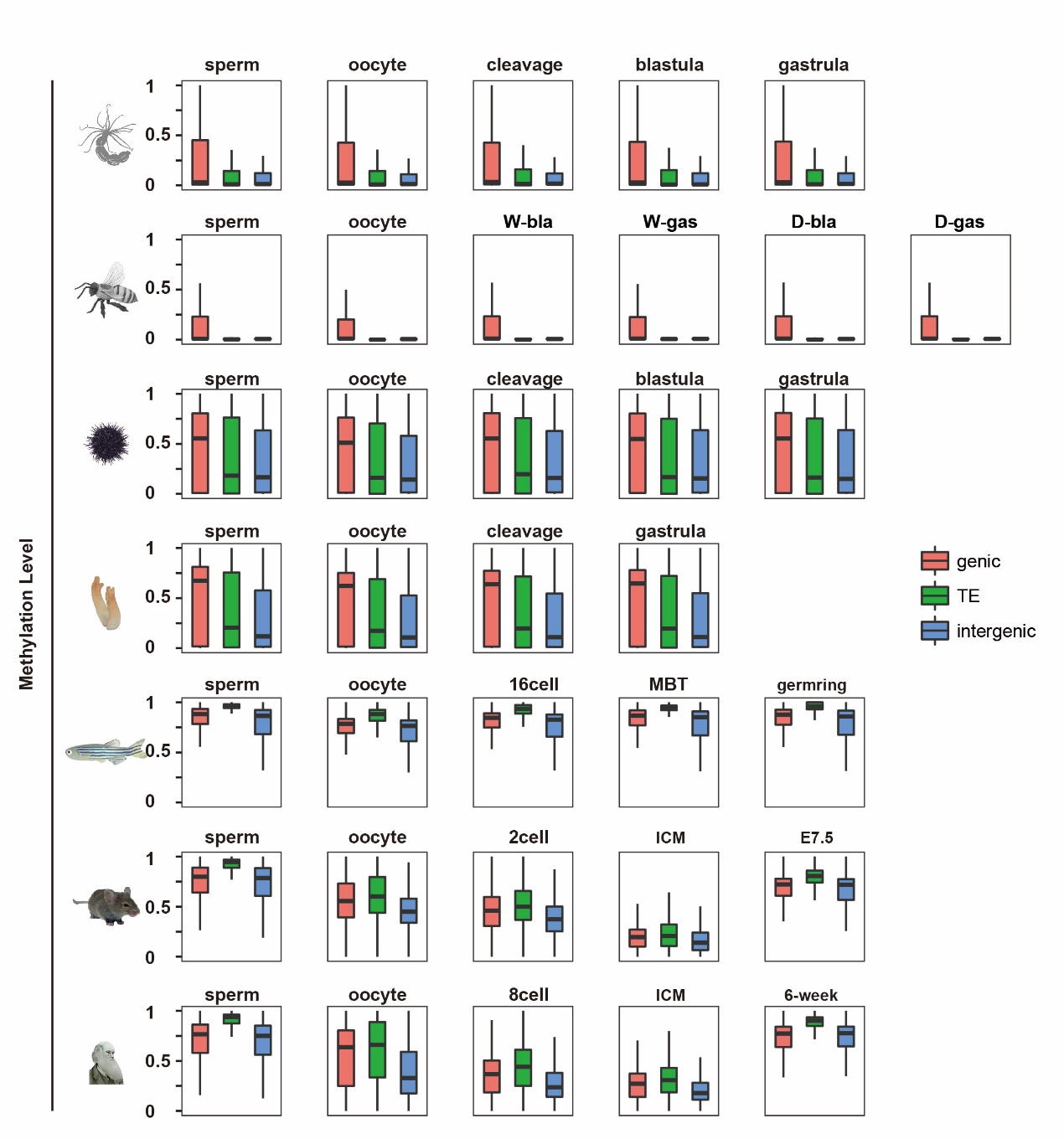


**Supplementary Fig. 2 |** **DNA methylation prefers to target genic regions in invertebrates.**

Box plots of average methylation levels of genic regions, transposable elements and intergenic regions in gametes and embryos across animals. Black bold lines indicates the median, edges the 25^th^/75^th^ percentile and whiskers the 2.5^th^/97.5^th^ percentile.


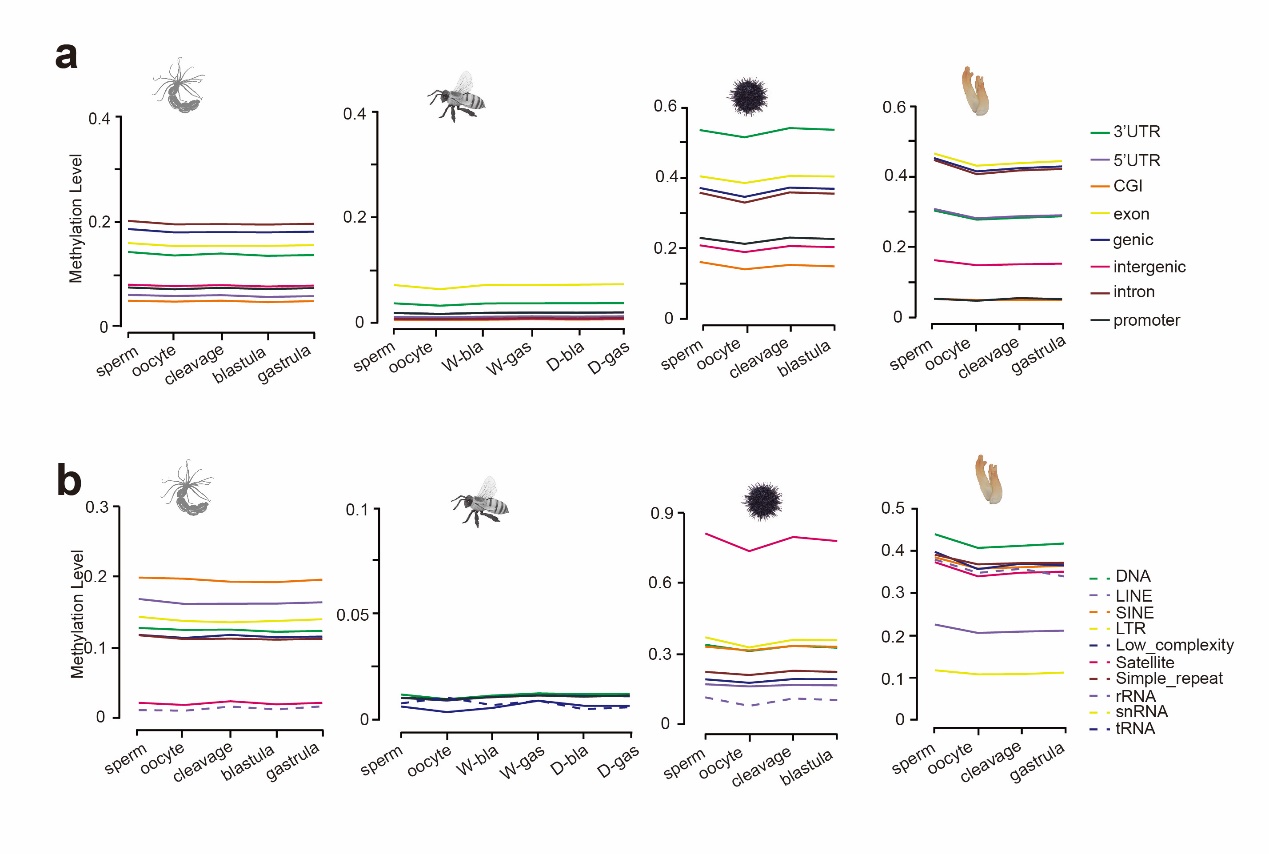


**Supplementary Fig. 3 |** **Dynamics of DNA methylation for different genomic elements**

**(a),** The dynamics of methylation levels of different genomic elements (genic related) during early embryogenesis. The average methylation is the mean value of the methylation levels of all CpGs located in the specific element. Florida sea anemone is using for the dynamic analyses. **(b),** The dynamics of methylation levels of different repeat elements.


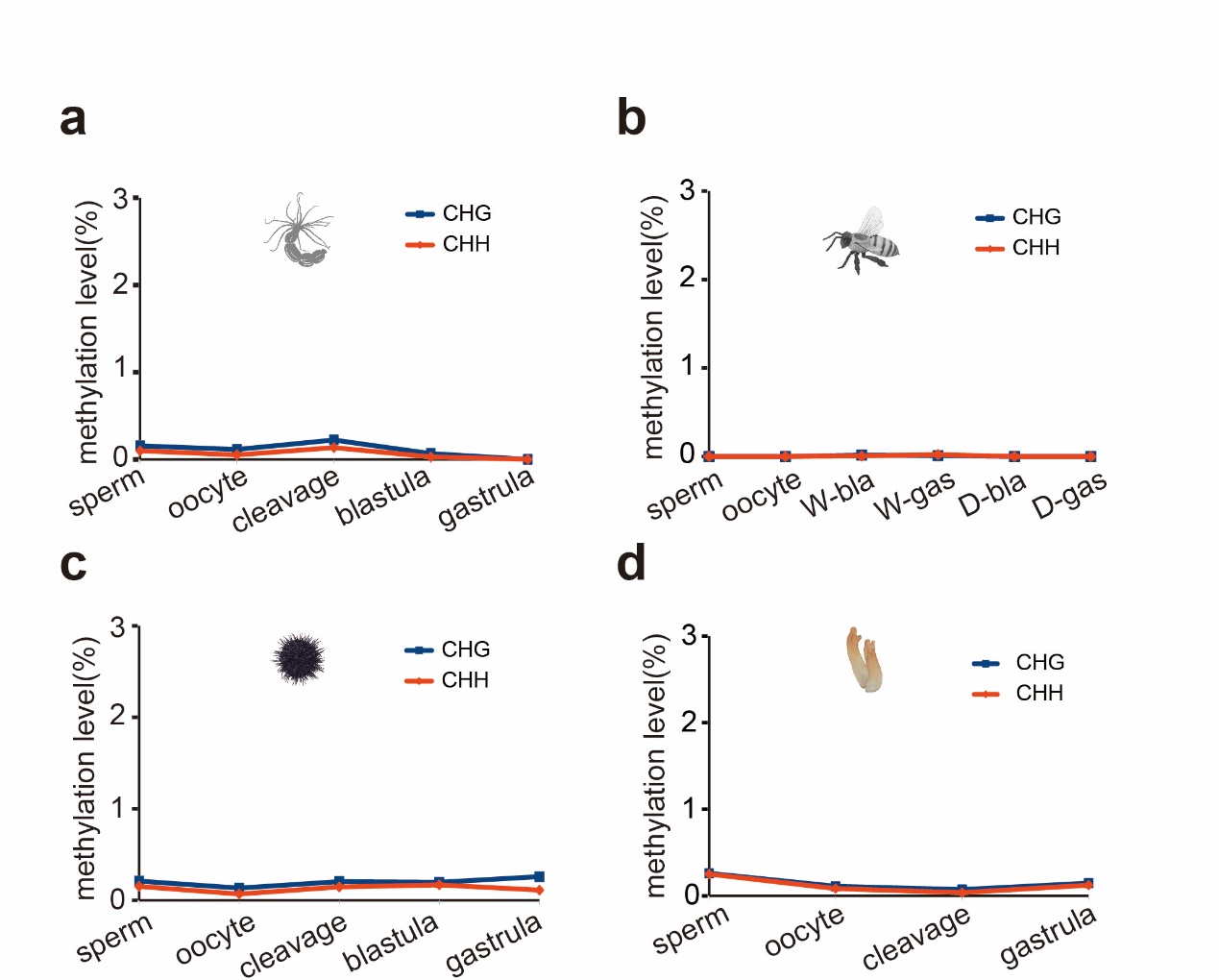


**Supplementary Fig. 4 | Non-CpG methylation in invertebrates**

**(a)-(d)**, Global methylation level of non-CpG in sea anemone**(a)**, honeybee**(b)**, sea urchin**(c)**, and ciona**(d)**, respectively.


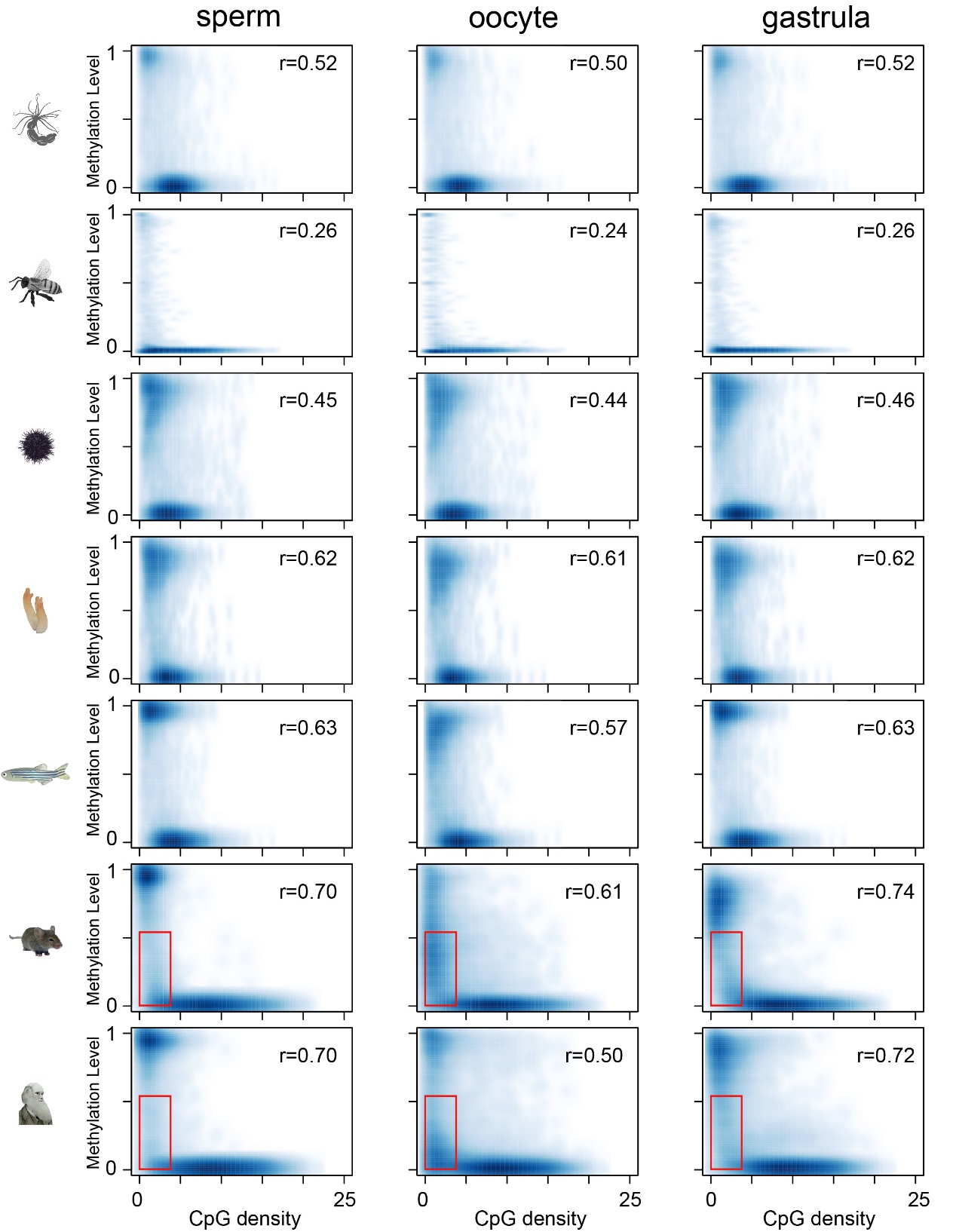


**Supplementary Fig. 5 | The scatterplots of DNA methylation levels and the CpG densities of promoter regions**

Scatterplots of CpG densities and methylation levels of promoter regions. The Pearson correlation coefficients (r) between CpG densities and DNA methylation levels across every sample were calculated and are included on the top right corner of each panel. Red boxes indicate promoters which have low CpG densities and low methylation levels.

**
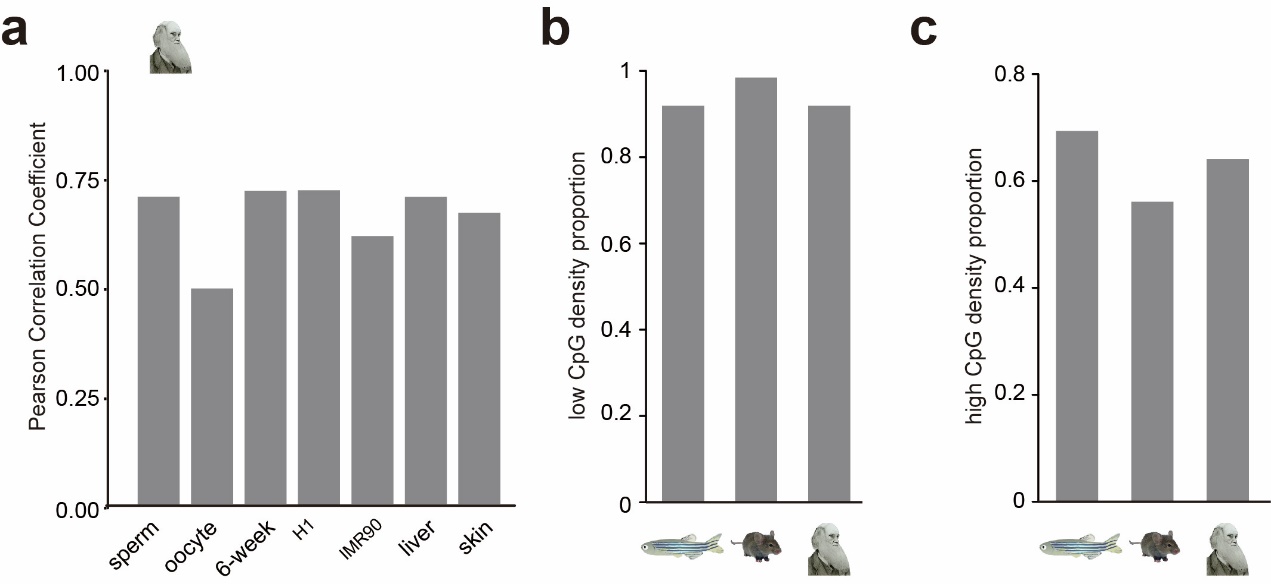
**

**Supplementary Fig. 6 | CpG density and DNA methylation.**

**(a),** Pearson correlation coefficient between CpG methylation level and CpG density in human tissues and cell types. **(b),** The proportion of low CpG density promoters among oocyte-specific hypomethylated promoters (versus sperm) in vertebrates. CpG density <=(3 CpGs / 100bp) is regarded as low CpG density. **(c),** The proportion of high CpG density promoters among oocyte-specific hypermethylated promoters (versus sperm) in vertebrates. CpG density > (3 CpGs / 100bp) is regarded as high CpG density.


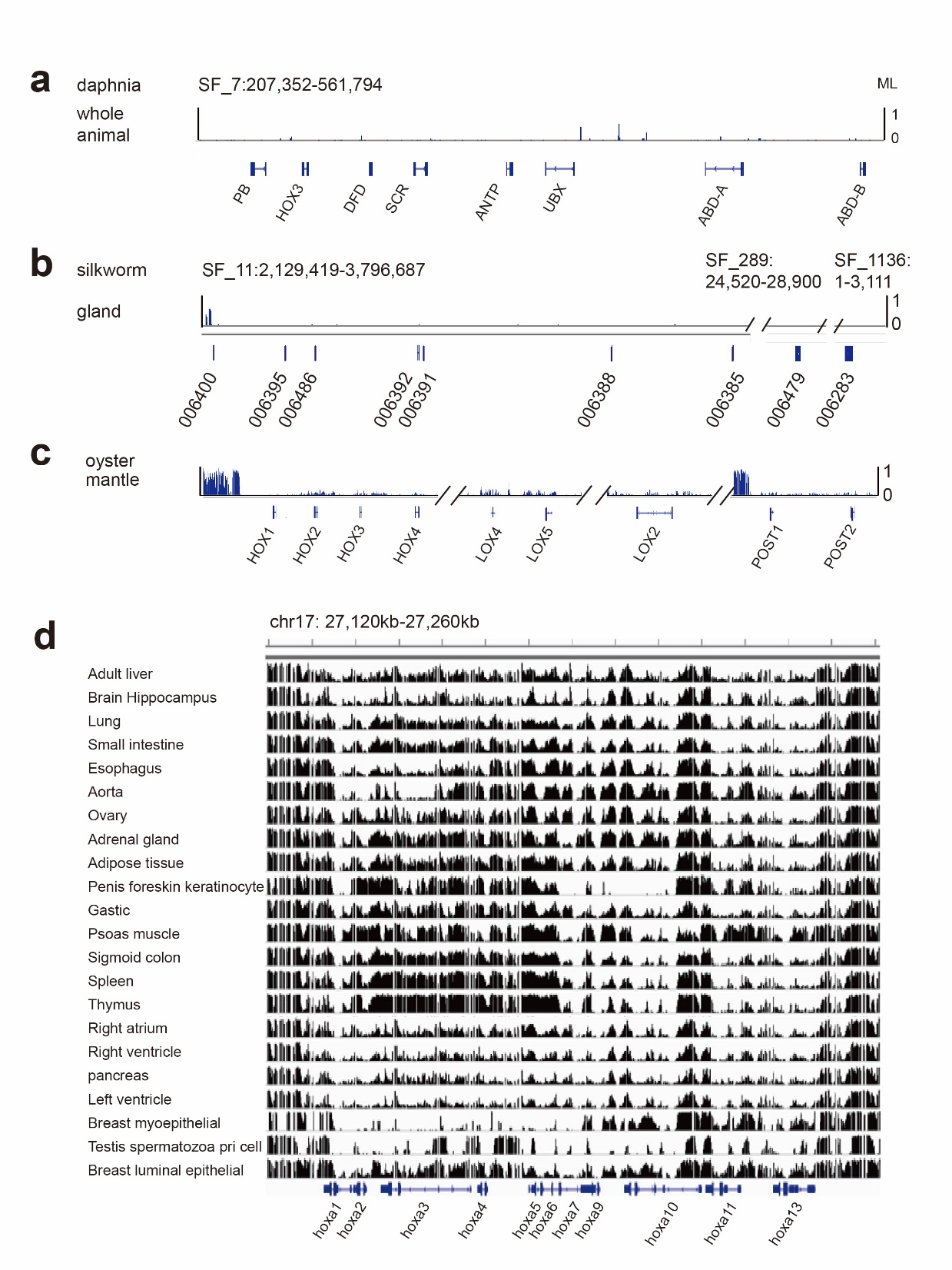


**Supplementary Fig. 7 | Methylation of HOX gene clusters of tissues in animals.**

**(a)**-(**c),** Genomic snapshots showing methylation at HOX gene clusters in daphinia, silkworm, and oyster, respectively. Oblique lines represent regions of HOX cluster that are non-contiguous or interrupted. The genomic coordinates of HOX gene fragments of oyster are in scaffold (SF) of SF_801:225,631-461,880, SF_145:664615-819314, SF_247:132,164-159,564, SF_1179:618,582-869,086, respectively. **(d),** Genomic snapshots showing methylation of HOXA gene cluster in different human organs.


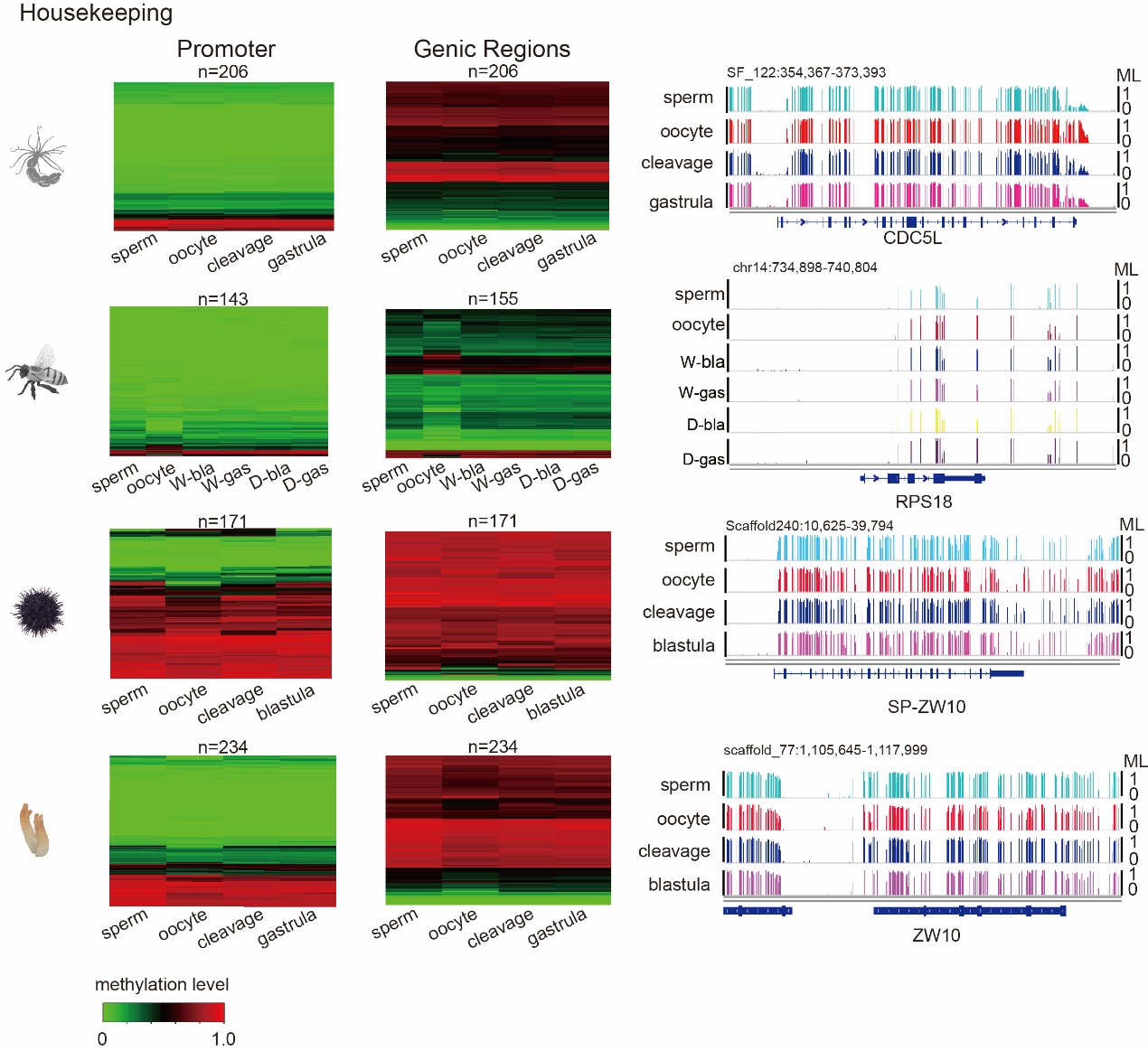


**Supplementary Fig. 8 | Reprogramming of housekeeping genes.** Heatmaps of the methylation reprogramming of promoters and genic regions of housekeeping genes from gametes to early embryos in different species. Unsupervised hierarchical cluster analysis was performed. “n” refers gene number. Genomic snapshots in the right panel show the methylation programming of housekeeping gene ZW10 or its paralog in different species.


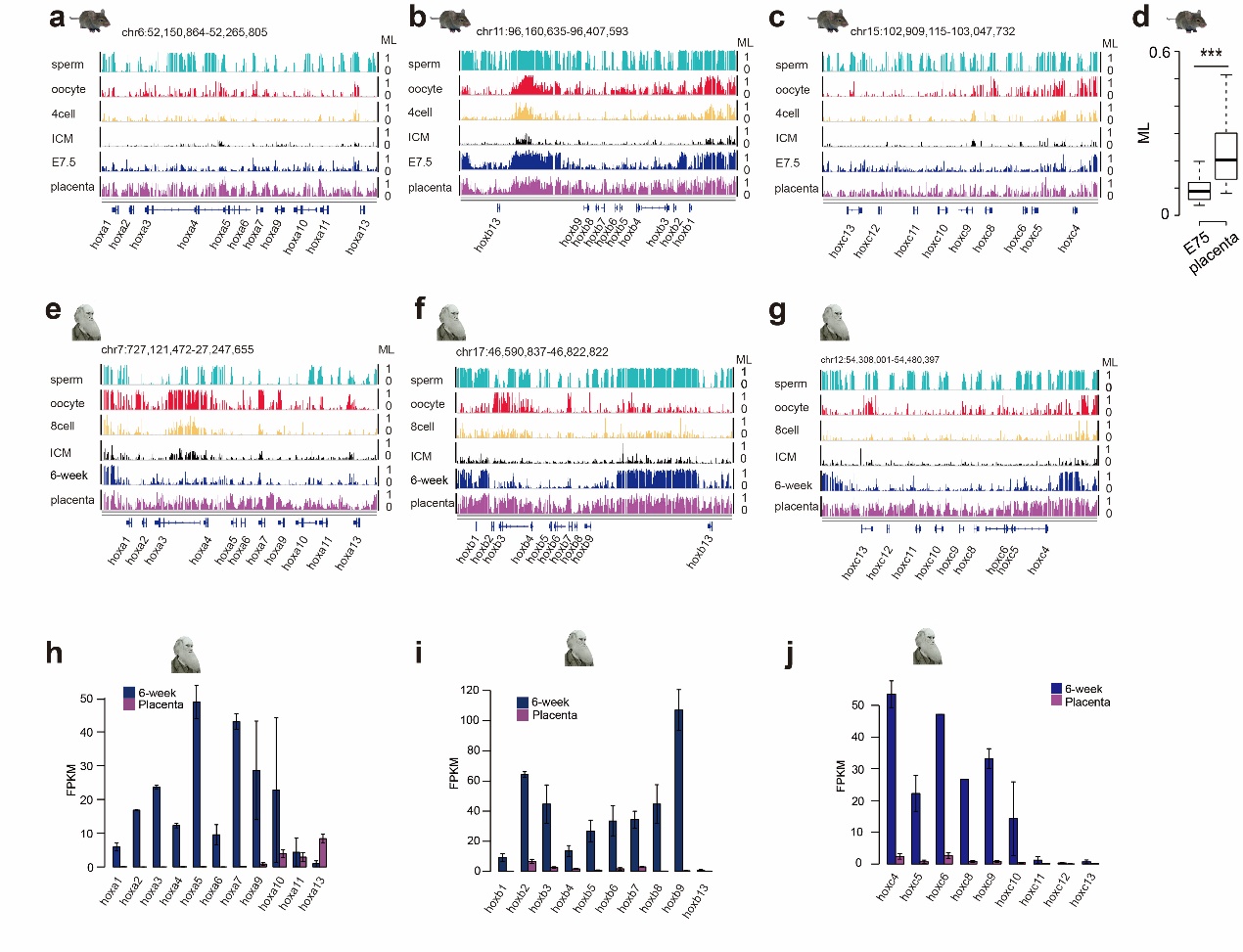


**Supplementary Fig. 9 | Methylation reprogramming and expression of HOX gene clusters. (a)**-(**c),** Genomic snapshots show methylation at three Hox gene clusters in mouse gametes, early embryos and placenta. **(d)**, Boxplots showing the methylation level differences between E7.5 embryos and placenta of HOX genes for promoter regions in mouse. P value was calculated by paired Wilcoxon signed-rank test. *** indicate *p* <0.001. **(e)-(g)**, Genomic snapshots show methylation at three HOX gene clusters regions in human gametes, early embryos and placenta. **(h)-(j)**, Normalized expression levels of HOX genes in human 6-week embryos and placenta.
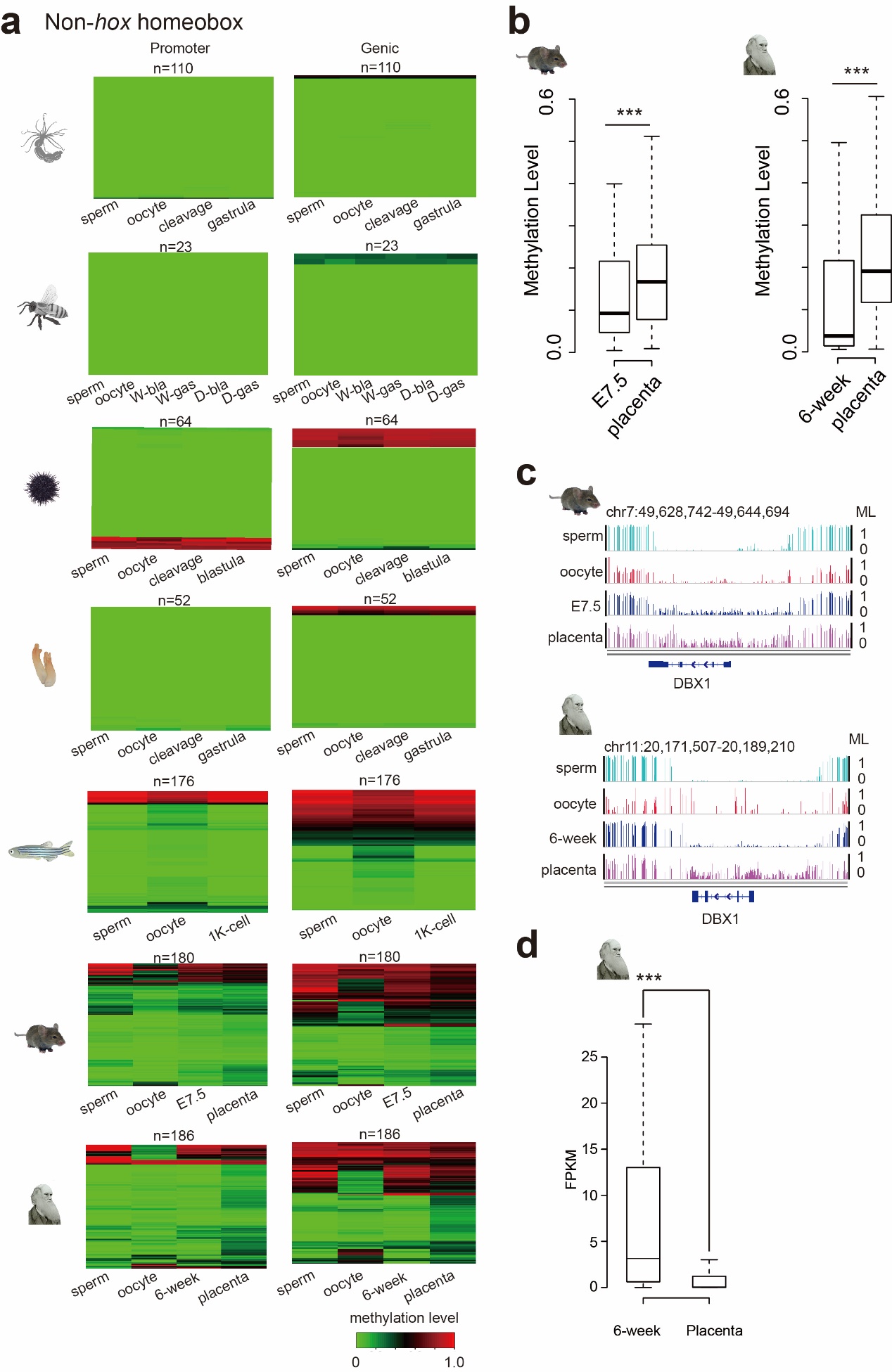


**Supplementary Fig. 10 | DNA methylation reprogramming and expression of non-Hox homeobox genes.**

**(a)**, Heatmaps of the methylation reprogramming of promoters and genic regions of non-Hox homeobox genes from gametes to early embryos for different species. **(b),** Boxplots showing the methylation level differences between 6-week or E7.5 embryos and placenta of non-HOX homeobox genes for promoter regions in mouse and human. P value was calculated by paired Wilcoxon signed-rank test. *** indicate *p* <0.001. **(c)**, Methylation reprogramming of a homeobox gene *DBX1* in oocytes, sperm, and E7.5/6-week embryos of mouse and human, respectively. **(d)**, Boxplots showing gene expression for non-Hox homeobox genes in human 6-week embryo and placenta. P value was calculated by paired Wilcoxon signed-rank test. *** indicate *p* <0.001.

**
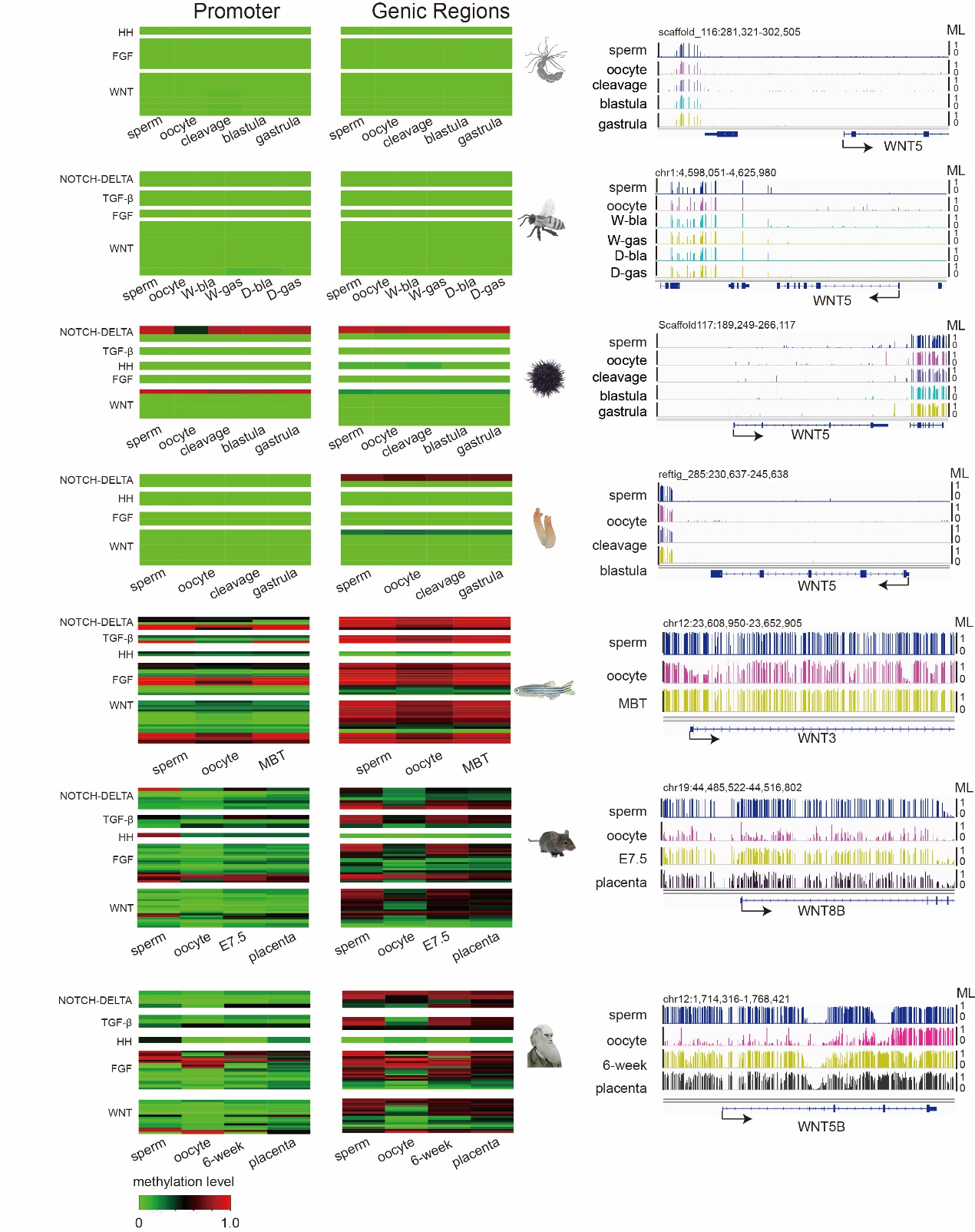
**

**Supplementary Fig. 11| Methylation of conserved signaling genes in different taxa.** Left panel, heatmaps of the average methylation level of promoter regions and genic regions of the signaling genes in sperm, oocytes and early embryos across different species. Unsupervised hierarchical cluster analysis was performed. Right panel, genomic snapshots displaying methylation dynamics of WNT genes in different species.

**
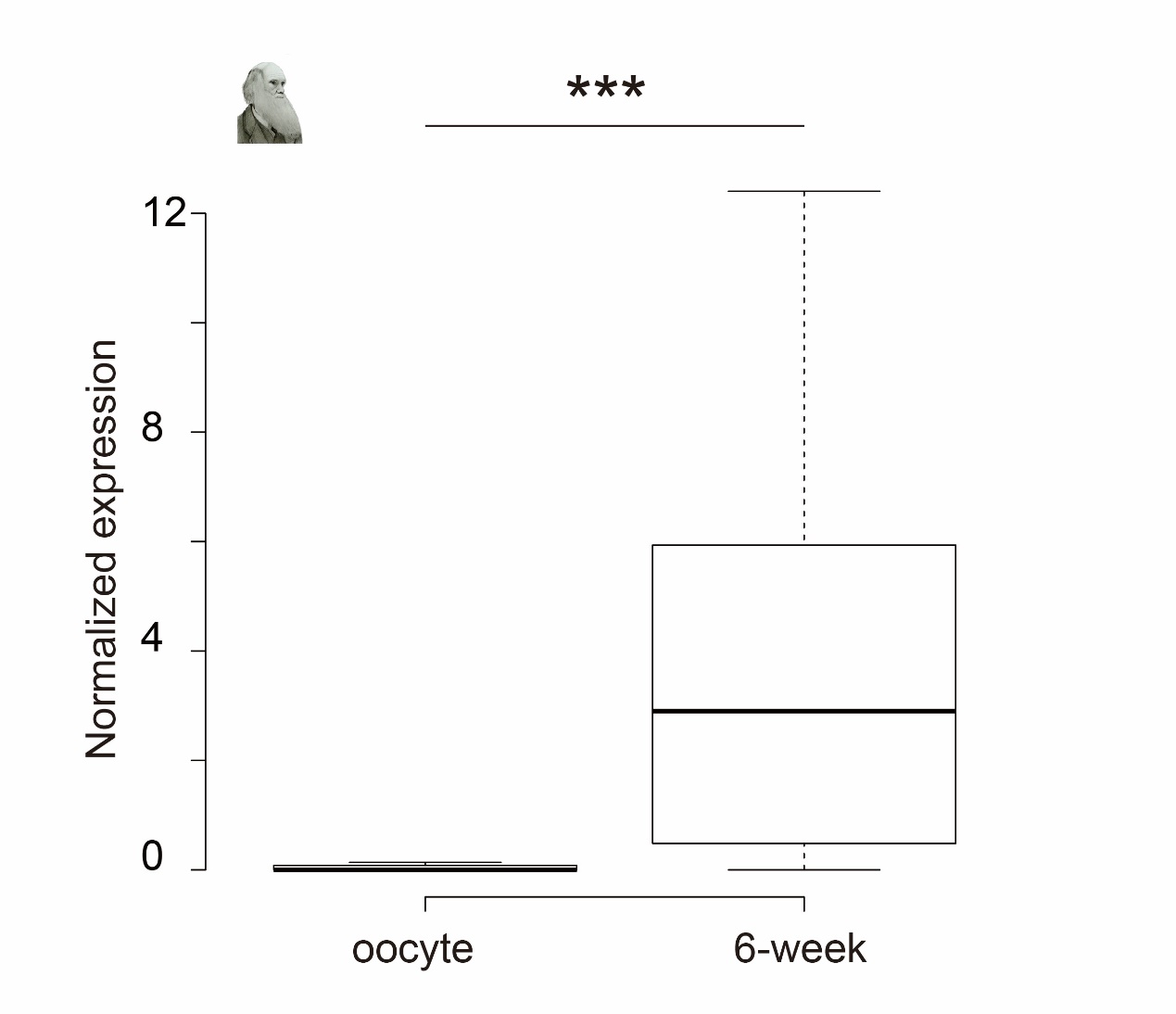
**

**Supplementary Fig. 12 | Expression of signaling genes in human.**

Boxplots showing gene expression for signaling genes in human oocytes and 6-week embryos. P value was calculated by paired Wilcoxon signed-rank test. *** indicate *p* <0.001.

**Table S1. Summary of Whole Genome Bisulfite Sequencing**

| species | stage | replicate | BSCR  (%) | Methylation Level | Coverage  (%) | depth |
| --- | --- | --- | --- | --- | --- | --- |
| Sea anemone | sperm | Fl | 99.2 | 0.108 | 87.88 | 31.85 |
|  |  | Ma | 99.1 | 0.122 | 84.89 | 9.56 |
|  | egg | Fl | 99.2 | 0.105 | 86.26 | 23.97 |
|  | cleavage | Fl | 99 | 0.104 | 87.3 | 15.16 |
|  |  | Ma | 99.1 | 0.133 | 79.1 | 7.34 |
|  | blastula | Fl | 99.3 | 0.105 | 89.5 | 40.99 |
|  |  | Ma | 99.1 | 0.129 | 81.33 | 10.67 |
|  | gastrula | Fl | 98.8 | 0.101 | 90.31 | 36.21 |
| Honey bee | sperm | rep1 | 99.3 | 0.007 | 97.47 | 47.85 |
|  |  | rep2 | 99.2 | 0.006 |  |  |
|  | oocyte | rep1 | 99.4 | 0.006 | 92.17 | 4.66 |
|  |  | rep2 | 99.1 | 0.005 |  |  |
|  | worker_ | rep1 | 99.3 | 0.007 | 97.3 | 21.68 |
|  | blastoderm | rep2 | 99.2 | 0.006 |  |  |
|  | drone_ | rep1 | 99.3 | 0.007 | 97.05 | 23.72 |
|  | blastoderm | rep2 | 99.2 | 0.007 |  |  |
|  | worker_ | rep1 | 99.1 | 0.006 | 98.28 | 59.23 |
|  | gastrulation | rep2 | 99.2 | 0.006 |  |  |
|  | drone_ | rep1 | 99.2 | 0.007 | 95.42 | 16.7 |
|  | gastrulation | rep2 | 99.2 | 0.007 |  |  |
| sea urchin | sperm | rep1 | 99.3 | 0.253 | 74.76 | 24.02 |
|  |  | rep2 | 99.3 | 0.267 |  |  |
|  | oocyte | rep1 | 99.2 | 0.224 | 71.76 | 9.59 |
|  |  | rep2 | 99.4 | 0.243 |  |  |
|  | cleavage | rep1 | 99.2 | 0.244 | 79.28 | 20.87 |
|  |  | rep2 | 99.4 | 0.256 |  |  |
|  | blastula | rep1 | 99.4 | 0.253 | 73.43 | 10.84 |
|  |  | rep2 | 99.3 | 0.251 |  |  |
|  | gastrula | rep1 | 99.4 | 0.249 | 71.27 | 8.31 |
|  |  | rep2 | 99.4 | 0.252 |  |  |
| sea squirt | sperm | rep1 | 99.4 | 0.284 | 99.19 | 64.16 |
|  |  | rep2 | 99.2 | 0.278 |  |  |
|  |  | rep3 | 99.3 | 0.287 |  |  |
|  | oocyte | rep1 | 99.5 | 0.264 | 98.46 | 40.57 |
|  |  | rep2 | 99.2 | 0.254 |  |  |
|  |  | rep3 | 99.2 | 0.259 |  |  |
|  | cleavage | rep1 | 99.5 | 0.26 | 99.38 | 84.09 |
|  |  | rep2 | 99.2 | 0.277 |  |  |
|  |  | rep3 | 99.5 | 0.279 |  |  |
|  | gastrula | rep1 | 99.5 | 0.272 | 98.21 | 55.11 |
|  |  | rep2 | 99.4 | 0.273 |  |  |

“BSCR” refers the bisulfite conversion rate (1- bisulfite non-conversion rate). “Fl” refers samples are sampled from Florida, and “Ma” refers samples are sampled from Massachusetts.

**Table S2. Pearson Correlation Coefficients between Replicates. (Excel file)**

**Table S3. GO Enrichment Analyses for Differentially Methylated Promoters. (Excel file)**

**References:**

1 Stefanik, D. J., Friedman, L. E. & Finnerty, J. R. Collecting, rearing, spawning and inducing regeneration of the starlet sea anemone, Nematostella vectensis. *Nature protocols* **8**, 916-923, doi:10.1038/nprot.2013.044 (2013).

2 Layden, M. J., Rottinger, E., Wolenski, F. S., Gilmore, T. D. & Martindale, M. Q. Microinjection of mRNA or morpholinos for reverse genetic analysis in the starlet sea anemone, Nematostella vectensis. *Nature protocols* **8**, 924-934, doi:10.1038/nprot.2013.009 (2013).

3 Fritzenwanker, J. H., Genikhovich, G., Kraus, Y. & Technau, U. Early development and axis specification in the sea anemone Nematostella vectensis. *Developmental biology* **310**, 264-279, doi:10.1016/j.ydbio.2007.07.029 (2007).

4 Paynter, E. *et al.* Flow cytometry as a rapid and reliable method to quantify sperm viability in the honeybee Apis mellifera. *Cytometry. Part A : the journal of the International Society for Analytical Cytology* **85**, 463-472, doi:10.1002/cyto.a.22462 (2014).

5 E.J., D. *The honeybee embryo*. 183-217 (Crowell Press, 1967).

6 Ronglin Yu, S. W. O. Early developmental processes in the fertilised honeybee (Apis mellifera) oocyte. *Journal of Insect Physiology* **45**, 763–767 (1999).

7 Stepicheva, N. A. & Song, J. L. High throughput microinjections of sea urchin zygotes. *Journal of visualized experiments : JoVE*, e50841, doi:10.3791/50841 (2014).

8 Christiaen, L., Wagner, E., Shi, W. & Levine, M. Isolation of sea squirt (Ciona) gametes, fertilization, dechorionation, and development. *Cold Spring Harbor protocols* **2009**, pdb prot5344, doi:10.1101/pdb.prot5344 (2009).

9 Jiang, L. *et al.* Sperm, but not oocyte, DNA methylome is inherited by zebrafish early embryos. *Cell* **153**, 773-784, doi:10.1016/j.cell.2013.04.041 (2013).

10 Wang, L. *et al.* Programming and inheritance of parental DNA methylomes in mammals. *Cell* **157**, 979-991, doi:10.1016/j.cell.2014.04.017 (2014).

11 Krueger, F. & Andrews, S. R. Bismark: a flexible aligner and methylation caller for Bisulfite-Seq applications. *Bioinformatics* **27**, 1571-1572, doi:10.1093/bioinformatics/btr167 (2011).

12 Smit, A. F. A. Interspersed repeats and other mementos of transposable elements in mammalian genomes. *Current Opinion in Genetics & Development* **9**, 657-663, doi:<http://dx.doi.org/10.1016/S0959-437X(99)00031-3> (1999).

13 Wu, H. *et al.* Detection of differentially methylated regions from whole-genome bisulfite sequencing data without replicates. *Nucleic Acids Research* **43**, e141-e141, doi:10.1093/nar/gkv715 (2015).

14 Huang, D. W., Sherman, B. T. & Lempicki, R. A. Systematic and integrative analysis of large gene lists using DAVID bioinformatics resources. *Nature protocols* **4**, 44, doi:10.1038/nprot.2008.211

<https://www.nature.com/articles/nprot.2008.211#supplementary-information> (2008).

15 Hosack, D. A., Dennis, G., Sherman, B. T., Lane, H. C. & Lempicki, R. A. Identifying biological themes within lists of genes with EASE. *Genome biology* **4**, 1-8, doi:10.1186/gb-2003-4-10-r70 (2003).
